# A new wearable multichannel magnetocardiogram system with a SERF atomic magnetometer array

**DOI:** 10.1038/s41598-021-84971-7

**Published:** 2021-03-10

**Authors:** Yanfei Yang, Mingzhu Xu, Aimin Liang, Yan Yin, Xin Ma, Yang Gao, Xiaolin Ning

**Affiliations:** 1 Hangzhou Innovation Institute, Beihang University, Hangzhou, 310051 China; 2 School of Instrumentation Science and Opto-Electronics Engineering, Beihang University, Beijing, 100191 China; 3 Department of Child Health Care Center, Beijing Children’s Hospital, Capital Medical University, National Center for Children’s Health, Beijing, 100045 China; 4 Research Institute for Frontier Science, Beihang University, Beijing, 100191 China; 5 Beijing Academy of Quantum Information Sciences, Beijing, 100193 China; 6 School of Physics, Beihang University, Beijing, 100191 China

**Keywords:** Engineering, Optics and photonics, Physics

## Abstract

In this study, a wearable multichannel human magnetocardiogram (MCG) system based on a spin exchange relaxation-free regime (SERF) magnetometer array is developed. The MCG system consists of a magnetically shielded device, a wearable SERF magnetometer array, and a computer for data acquisition and processing. Multichannel MCG signals from a healthy human are successfully recorded simultaneously. Independent component analysis (ICA) and empirical mode decomposition (EMD) are used to denoise MCG data. MCG imaging is realized to visualize the magnetic and current distribution around the heart. The validity of the MCG signals detected by the system is verified by electrocardiogram (ECG) signals obtained at the same position, and similar features and intervals of cardiac signal waveform appear on both MCG and ECG. Experiments show that our wearable MCG system is reliable for detecting MCG signals and can provide cardiac electromagnetic activity imaging.

## Introduction

Cardiac electrical activity produces electrical potentials on the body surface, which are of great physiological and clinical importance. An electrocardiogram (ECG) is usually used as the primary diagnostic tool in cardiology. However, the detection and identification of regional electrical events in the heart needs a record of the potential distribution over the entire chest, which is not possible with conventional ECG^1^. Therefore, a multichannel technique called body surface potential mapping (BSPM) has been widely studied as an alternative to conventional ECG^2^. BSPM is sensitive in detecting local electrical events, and it provides a high spatial resolution.

A close relationship exists between the electric current and magnetic field. Unlike cardiac electrical signals, the permeability of the human body is constant, and magnetic signals are barely affected by the inhomogeneous conductivity of bodily tissues, making them more reliable for the detection of biological phenomena^3^. Due to the different physical characteristics between electric and magnetic fields, cardiac magnetic field signals may provide information on cardiac current that is difficult to obtain by ECG or BSPM. For example, cardiac magnetic field signals are sensitive to ‘tangential’ and vortex current sources, while electrical signals are more sensitive to ‘radial’ sources^4^. The magnetocardiogram (MCG) was introduced as a comparatively sensitive technique in the 1970s as the magnetic equivalent of the ECG^5^. Then, in the 1990s, multichannel MCG systems appeared as a magnetic equivalent to BSPM. MCG has been applied in the diagnosis of cardiac diseases such as cardiac ischemia, arrhythmias, and fetal heart diseases^6–8^. The combination of MCG and BSPM leads to better source estimates^2,4^. The combined MCG and BSPM can also provide comprehensive data for a comparison of electric and magnetic field properties under physiological conditions^9^.

However, conventional multichannel MCG systems based on SQUID (superconducting quantum interference device) magnetometers are not widely used in hospitals^10^. The clinical application is hindered by their large size and high cost^11^. Recently, a highly sensitive room temperature optically pumped magnetometer (OPM) has attracted significant attention^12–15^. The advantage of not requiring the Dewar for cryogenic cooling liquid makes system miniaturization possible and improves the flexibility of the arrangement of the sensor array for multichannel measurement. The most commonly used OPMs in MCG systems include scalar OPMs, such as Mx magnetometers^16,17^, and vector OPMs, such as spin exchange relaxation-free (SERF) magnetometers^18,19^. Vector magnetometers can measure multiple components of the magnetic field and thus can obtain more complete information about the field and provide much better localization information for the detection of cardiac anomalies^20^.

Several multichannel MCG systems based on OPMs have been developed in recent years. In 2009, Bison et al.^21^ presented a MCG imaging system based on a grid of 19 Mx magnetometers over the chest. However, as the scalar Mx magnetometer only measures the magnitude of the magnetic field, it is insensitive to the direction of the magnetic field, which poses problems for source localization and related applications^19^. In 2012, Kamada et al.^3^ asynchronously acquired 25-channel human MCGs using a SERF potassium atomic magnetometer, and the MCG maps agree well with those measured by SQUID magnetometers. In 2012, Wyllie et al.^18^ presented a portable four-channel SERF atomic magnetometer array for MCG measurement. The SERF magnetometer array is mounted on an existing SQUID gantry. The minimum planar array spacing for all four elements is 4.5 cm. In 2019, a commercial cardiac imaging platform using SERF OPMs from Genetesis was developed. Inc.^22^ received FDA (Food and Drug Administration, US) 510(k) clearance.

However, in all of the systems mentioned above, sensors are fixed in a gantry or a bed, making it not flexible enough to change the sensor distribution. A convenient and wearable sensor array is required for a multichannel MCG system. This is especially true for fetal MCG (fMCG) measurement, as the size and shape of a pregnant woman's abdomen vary greatly during pregnancy, and a fixed shape limits the flexibility of placing sensors in the optimal position of the pregnant woman's abdomen. For this purpose, an fMCG system based on atomic magnetometers was developed in 2015^23^. Twenty-five individual microfabricated OPMs are inserted into three flexible belt-shaped holders and assembled into a conformal array. However, due to the inconvenience of performing physical activity during acquisition, a wearable MCG system should be developed.

In some existing MCG systems, multichannel MCG detection is realized by sequential scanning. The measurement is time consuming, and some transient information on cardiac activity is omitted. This time-consuming measurement is usually incapable of capturing transient cardiac activity. As MCG imaging produced by asynchronous measurement is not accurate enough, simultaneous MCG imaging would be more reliable.

In this study, a wearable multichannel MCG system based on a SERF atomic magnetometer array is developed. The system consists of a magnetically shielded device, a wearable SERF magnetometer array, and a computer for data acquisition and processing. Multichannel MCG signals of a healthy subject are obtained to demonstrate the technical feasibility of the system. Simultaneously, the ECG signal is recorded as the reference for cross validation. The distribution of MCG signals is visualized by magnetic field maps (MFM) and pseudocurrent density (PCD) maps after preprocessing.

## Results

### System components

**Figure 1:**
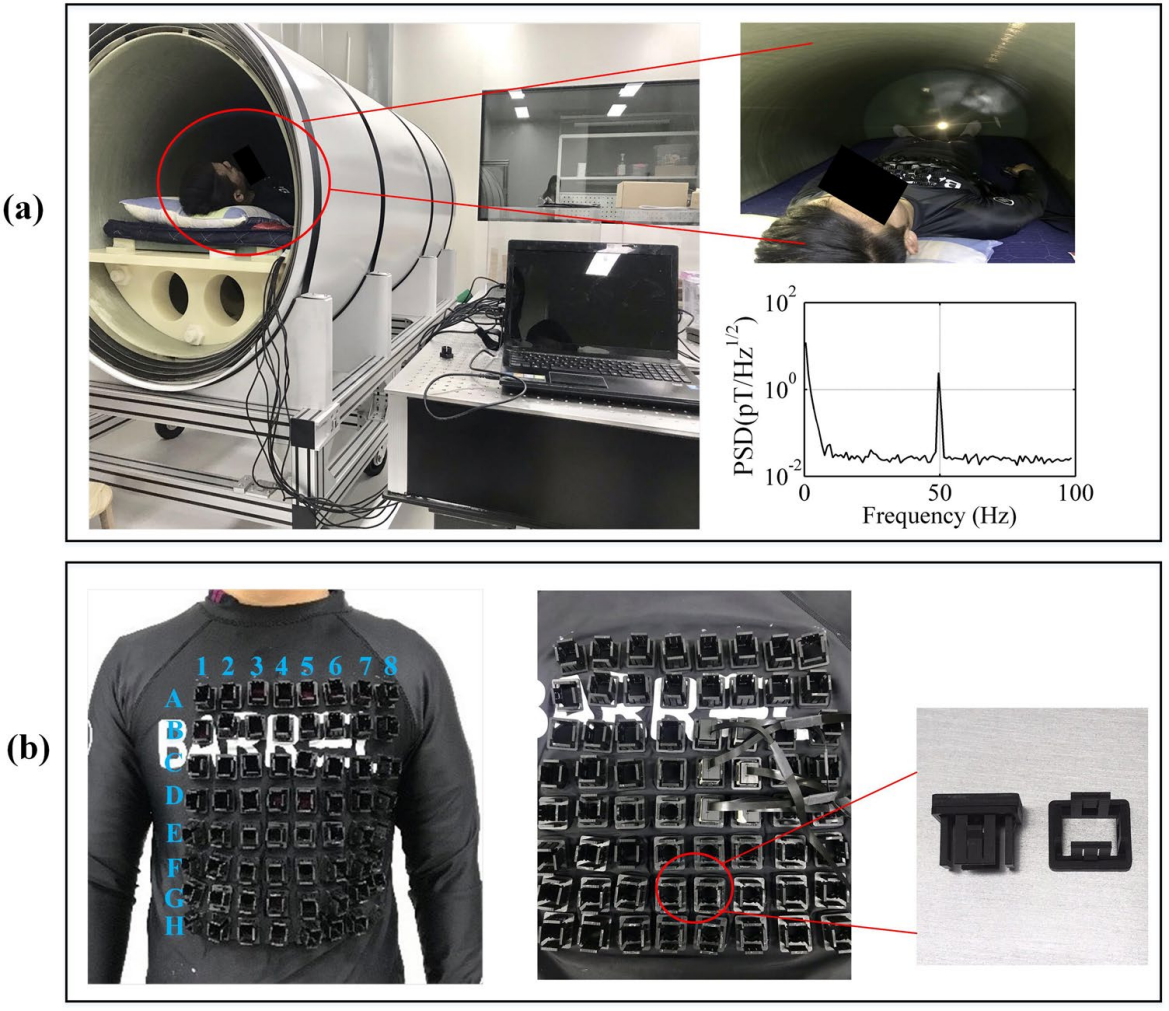
(**a**) From left to right is the magnetically shielded cylinder and magnetic noise spectrum diagram when the shielded cylinder is closed. The magnetic shield is closed during the process of acquiring the MCG signal. (**b**) Position labels of the 8 × 8 array with 30 mm intervals, QZFM magnetometer and customized receptacles.

The system consists of a magnetically shielded device, a wearable SERF magnetometer array, and a data acquisition and processing computer, as shown in Fig. 1a. The SERF magnetometer is among the most sensitive magnetic detectors and operates in low field environments; hence, a magnetically shielded device is necessary. A magnetically shielded room (MSR) is generally used. Since the size of the SERF magnetometer is much smaller than that of the SQUID magnetometer, a magnetically shielded cylinder^22^ is also sufficient. In this study, a person-sized magnetically shielded cylinder made of four-layer permalloy and one-layer aluminum is used to reduce the interference of external magnetic fields. The static shielding factor is 10^4^, and the residual magnetic field inside the magnetically shielded cylinder is under 5 nT when closed.

The SERF magnetometers employed here are produced by QuSpin Inc^24^. The size of each sensor is 12.4 × 16.6 × 24.4 mm, and it is connected to the control electronics module by a 6.5 m cable. The noise level specified by the vendor is 7–10 fT/√Hz. Normal operation requires the background noise to be less than 50 nT. The operational dynamic range is ± 5 nT. Each sensor is a separate unit containing all the necessary optical components, including a 795-nm semiconductor laser, an optical device for laser beam modulation, an 87Rb vapor cell and a photodetector. A laser diode produces light that is tuned to the resonance frequency of the 87Rb atoms. The light beam is collimated and directed to pass through the vapor cell, which has been heated to approximately 150 °C, and then directed onto a photodetector. The magnetic compensation coil is powered on to compensate for the background magnetic field. When the background magnetic field is equal to zero, the rubidium atoms become largely transparent. Subtle changes in the magnetic field can change the intensity of transmitted light, which is detected by the photodetector.

Different from previous MCG systems, the magnetometer array is wearable in this system. A customized wearable measurement device is employed, as shown in Fig. 1b. A kind of tailor-made receptacle is designed and produced by ABS plastic using 3D printing technology, whose size is matched with the magnetometer sensor head. A close-fitting swimwear is used to mount the receptacles. The measurement points are marked on the swimwear, and then small holes are punched, centered on the measurement points. The receptacles are mounted on the swimwear perpendicularly by a socket across the holes. SERF magnetometers are plugged into the receptacles to obtain multichannel MCG signals. The Z-axis of SERF magnetometers is perpendicular to the surface of the thorax, and the Z-axis outputs represent the normal components of the cardiac magnetic field. The position of each magnetometer is indicated as the position of the corresponding marked point on the swimwear.

A data acquisition (DAQ) device is needed for digitizing the analog output of the magnetometers. A 32-channel commercial DAQ device is used, including a chassis (PXIe-1071, National Instruments, US) and two 16-channel acquisition boards (PXIe-4499, National Instruments, US), which can receive the outputs of 32 magnetometers at the same time at most. It is controlled by customized LabVIEW (National Instruments, US) software. The resolution of the DAQ device is 16 bits, and the total sampling rate is 250 kS/s. The acquisition board we used has a low-pass filter to filter out high-frequency noise above 10 kHz. A computer is necessary for data acquisition and processing.

### Comparison between MCG and ECG

**Figure 2:**
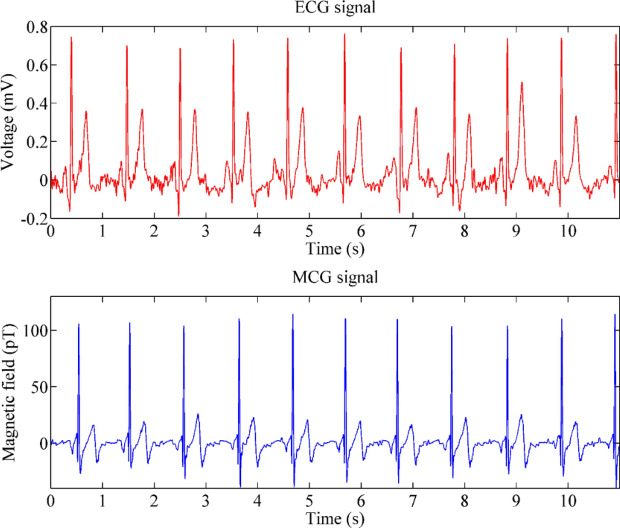
From top to bottom are ECG signals and MCG signals processed by digital filtering. The y-axis represents the magnetic field strength in units of pT, and the x-axis represents the sampling time in units of s.

To verify the measured MCG signals, MCG and ECG signals recorded at the same position (D4) are compared, as shown in Fig. 2, which are filtered using the digital filter. The baselines of raw signals are corrected using a median filter with a window length of 601 ms. Then, the high-frequency interferences are reduced using a 45 Hz low-pass filter. Similar waveform features can be seen in both signals. The typical features, i.e., the P waves, QRS complex and T waves, can be clearly distinguished. The PR, QRS and QT intervals of both MCG and ECG are also highly consistent. MCG is mainly sensitive to intra- and extracellular activation currents, whereas the chest leads of an EGG (or BSPM) measure the potential differences mainly generated by the secondary (volume) current flowing just below the skin^5^. This is the main reason for the slight difference between the two types of signals. It was verified that MCG signals measured by the system are valid.

### Signal processing and MCG imaging

**Figure 3:**
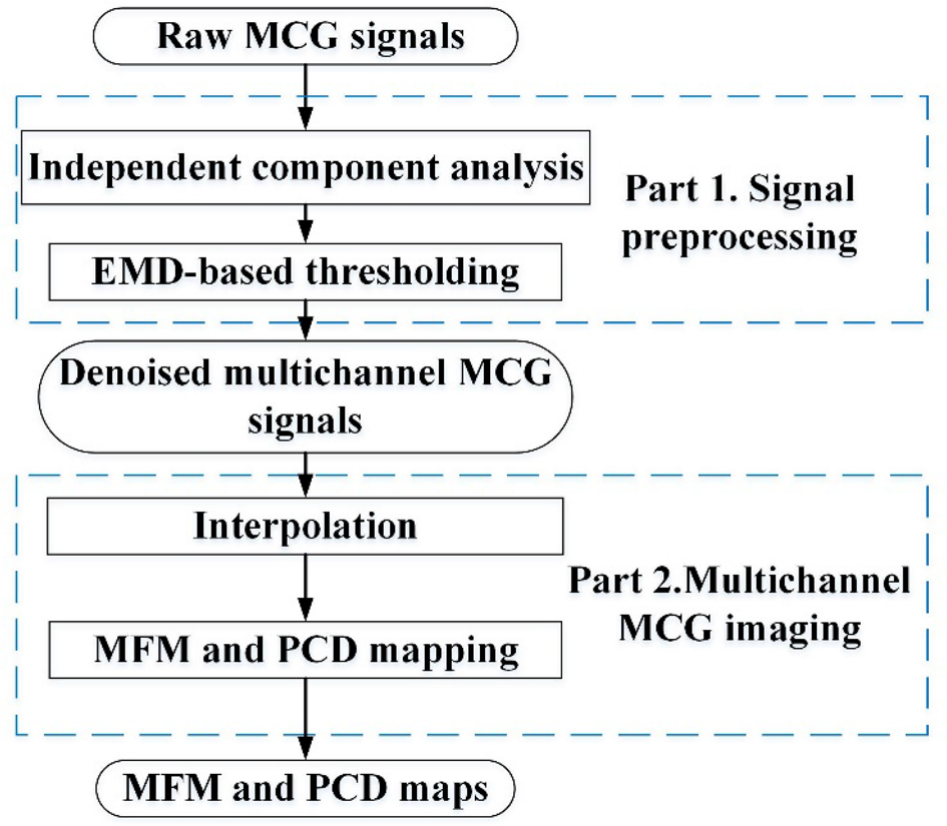
Diagram of the MCG signal processing and imaging method. Signal processing: independent component analysis and empirical mode decomposition (EMD). After processing the signal, through cubic spline interpolation, the representation is magnetic field maps (MFM) and pseudocurrent density (PCD) maps.

After multichannel MCG signals are acquired, signal processing and imaging are carried out. Figure 3 shows the block diagram of the multichannel MCG signal processing and imaging. Raw MCG signals are often contaminated with environmental noise, baseline drift, respiratory interference, and power line noise, which should be removed before imaging.

**Figure 4:**
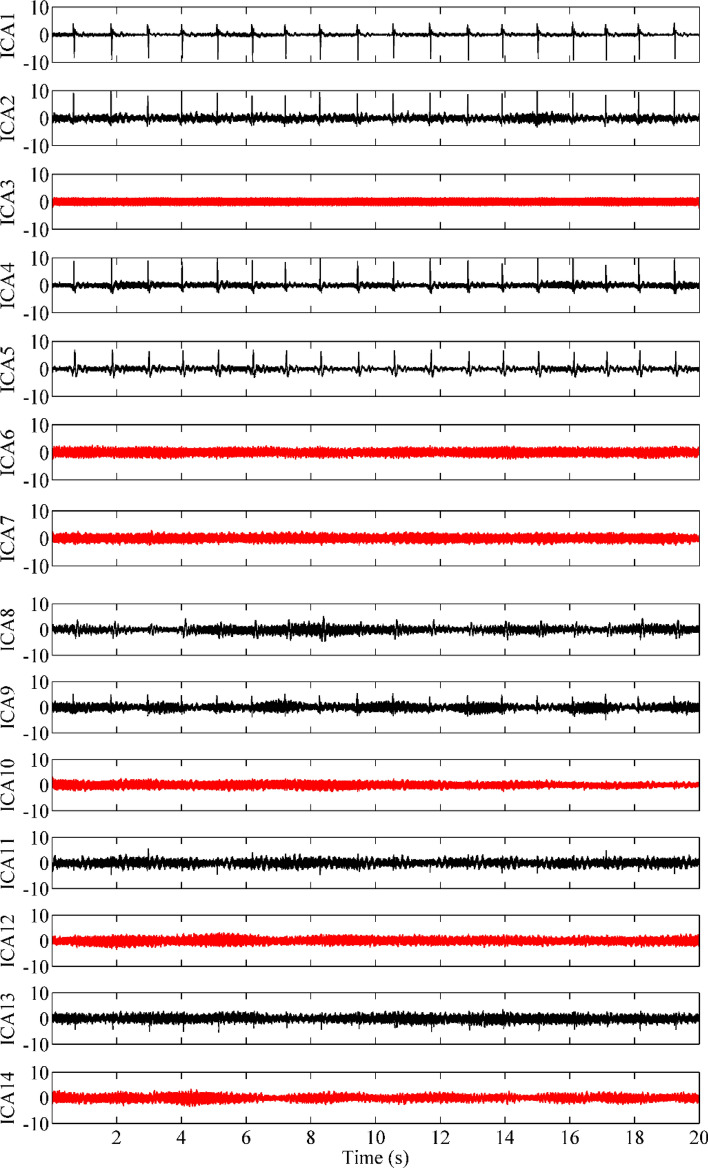
The ICs obtained from the ICA for the MCG data. Artifact components for visual recognition (50 Hz and harmonics) are represented in red, and nonartifact components (guided by cardiac periodic features such as QRS and T waves) are represented in black.

To reduce the interference of noise, raw signals are processed using the method for independent component analysis (ICA) and empirical mode decomposition (EMD). Principal component analysis (PCA) decomposition was performed on the original multichannel MCG signals, and 99% of the signal power was taken as the signal subspace, while the remaining 1% was ignored as the noise subspace. For the data in this paper, of the 20 components, 14 components were found to satisfy 99% of the total signal power. Figure 4 shows that the multichannel MCG data were decomposed into 14 ICs by ICA. The kurtosis values of 14 ICs are calculated. When the kurtosis value is less than three, we consider the corresponding IC (IC3, IC6, IC7, IC10, IC12, IC14) to be the noise component and set it to zero. In Fig. 4, the IC marked with the red line is the noise IC, and the remainder is the useful IC.

**Figure 5:**
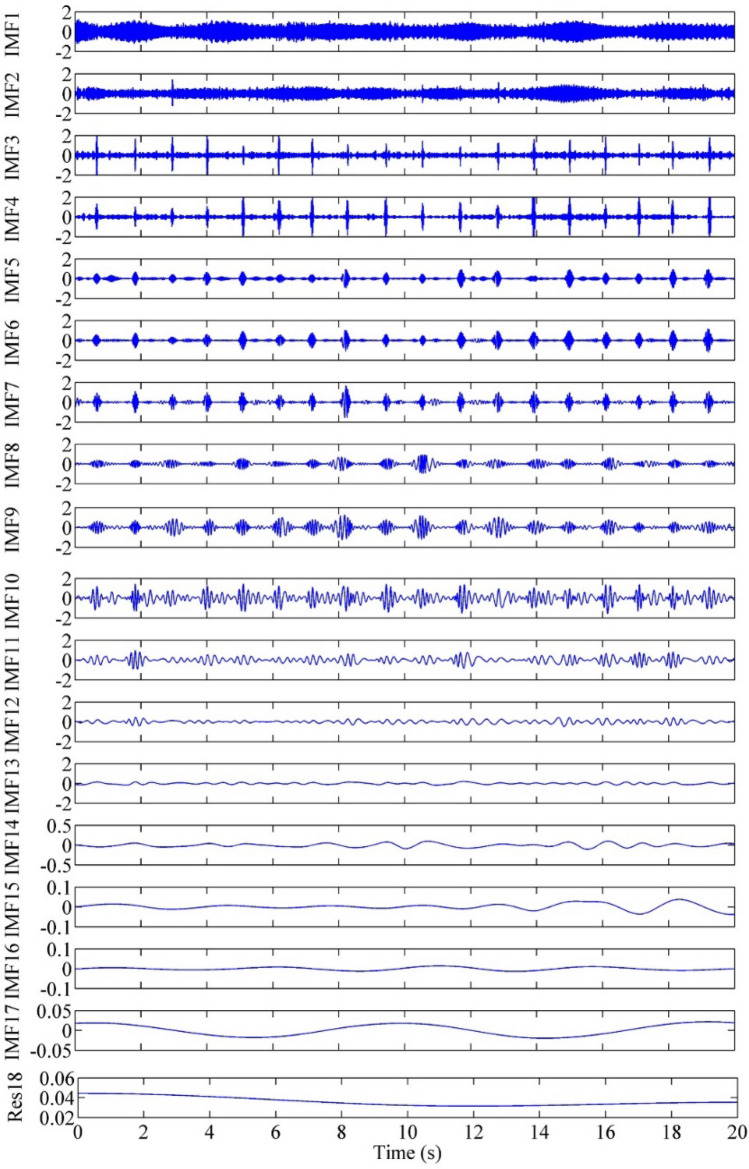
EMD decomposition IMFs of IC2.

**Figure 6:**
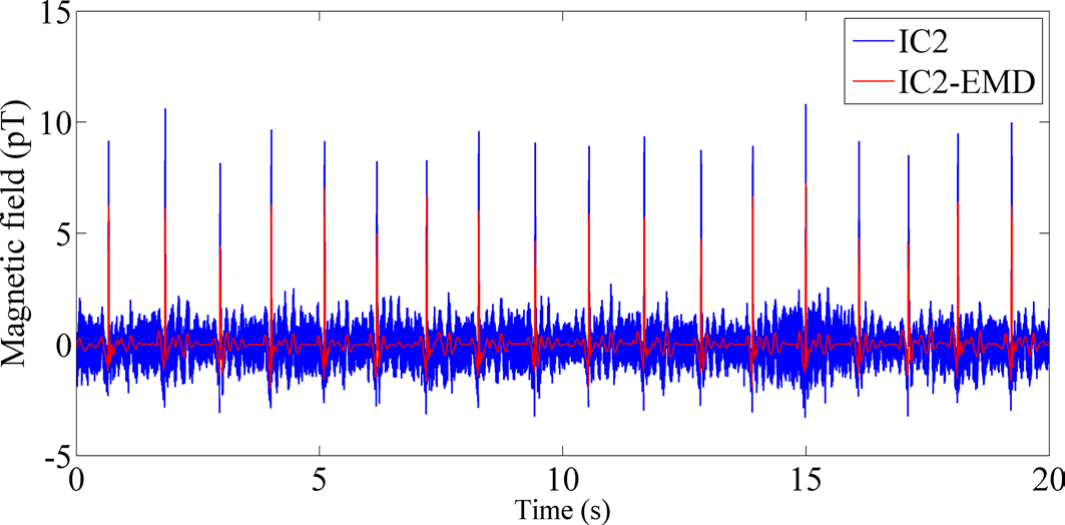
The blue line represents IC2, and the red line represents IC2 denoised by EMD.

Then, each useful IC was decomposed based on EMD, and each IMF was processed with a segmented threshold. As an illustration, IC2 was decomposed by EMD, as shown in Fig. 5. According to the decomposed IMFs, the corresponding evaluation parameters are calculated, threshold processing is carried out, and the denoised IC is reconstructed. Figure 6 shows the comparison of IC2 and IC2 denoised by EMD. The residual noise contained in IC2 is effectively removed.

**Figure 7:**
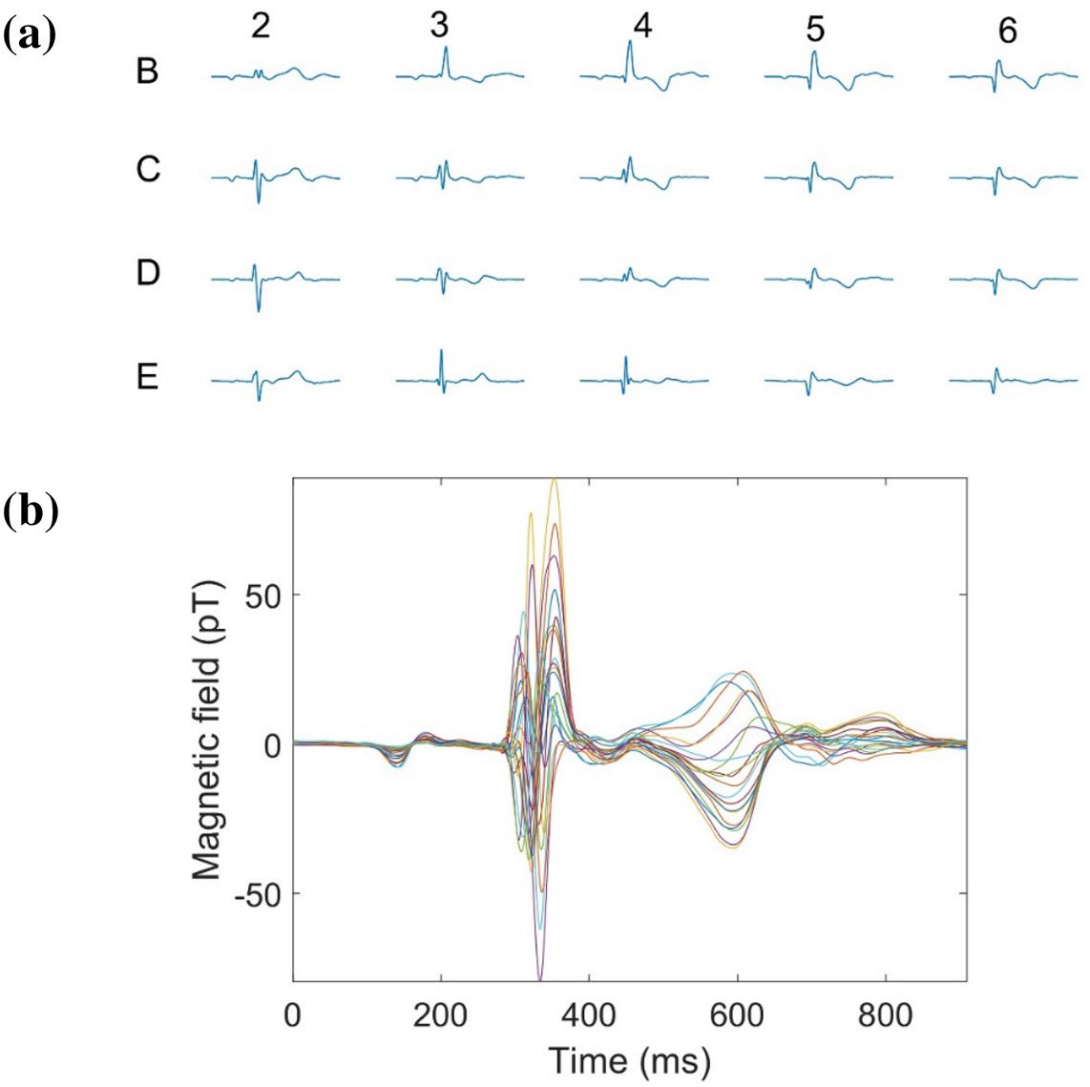
(**a**) The MCG preprocessed by ICA-EMD at the 20 spatial locations for a single epoch (unaveraged). (**b**) Butterfly of MCG.

All of the processed 20-channel MCG signals within a cardiac cycle are plotted by their position labels, as shown in Fig. 7a. All MCG signals are plotted together in Fig. 7b to show the temporal distribution of the 20-channel MCGs. Due to the different electrophysiological activities of the heart at different locations, the MCG waveforms corresponding to different locations are different. However, it can be seen from the butterfly diagram that normal MCG has the characteristics of the P wave, QRS wave and T wave groups.

**Figure 8:**
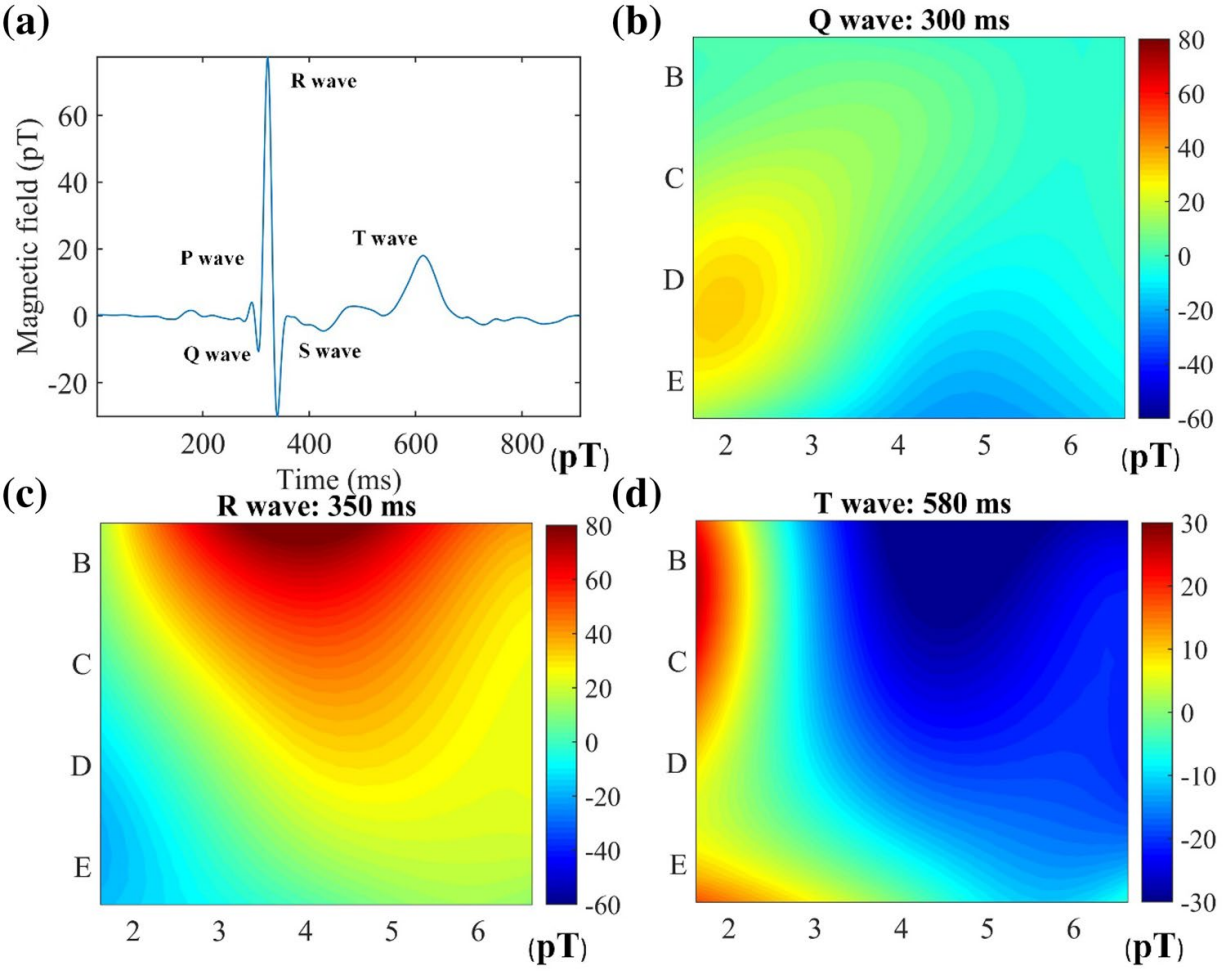
(**a**) MCG signal at D2. The MCG signal has the characteristics of a P wave, QRS wave and T wave group. Twenty-channel MFMs of the (**b**) Q peak, (**c**) R peak and (**d**) T peak. The Y axis and X axis coordinates correspond to the position of the sensor, as shown in Fig. 1. Normal MFM has a single dipole structure. The color depth of the figure represents the intensity of the magnetic field, corresponding to the color bar in the figure.

Figure 8 shows normal MFMs for the Q peak (300 ms), R peak (350 ms), and T peak (580 ms) in a cardiac cycle to display the magnetic field distribution of the heart. Temporal variations in cardiac magnetic field distribution can be observed. The MFMs correspond to the distribution of the sensor on the trunk surface. MFMs show that the cardiac magnetic field of a healthy subject is a dipolar field, which agrees well with the results of previous works^3,25^. During the Q-wave to R-wave period, the positive dipole of the MFMs moves from the right ventricle position to the left ventricle position, representing the process of ventricular depolarization. In the T-wave period, the negative pole is located in the left ventricle, which represents the repolarization of the ventricle.

**Figure 9:**
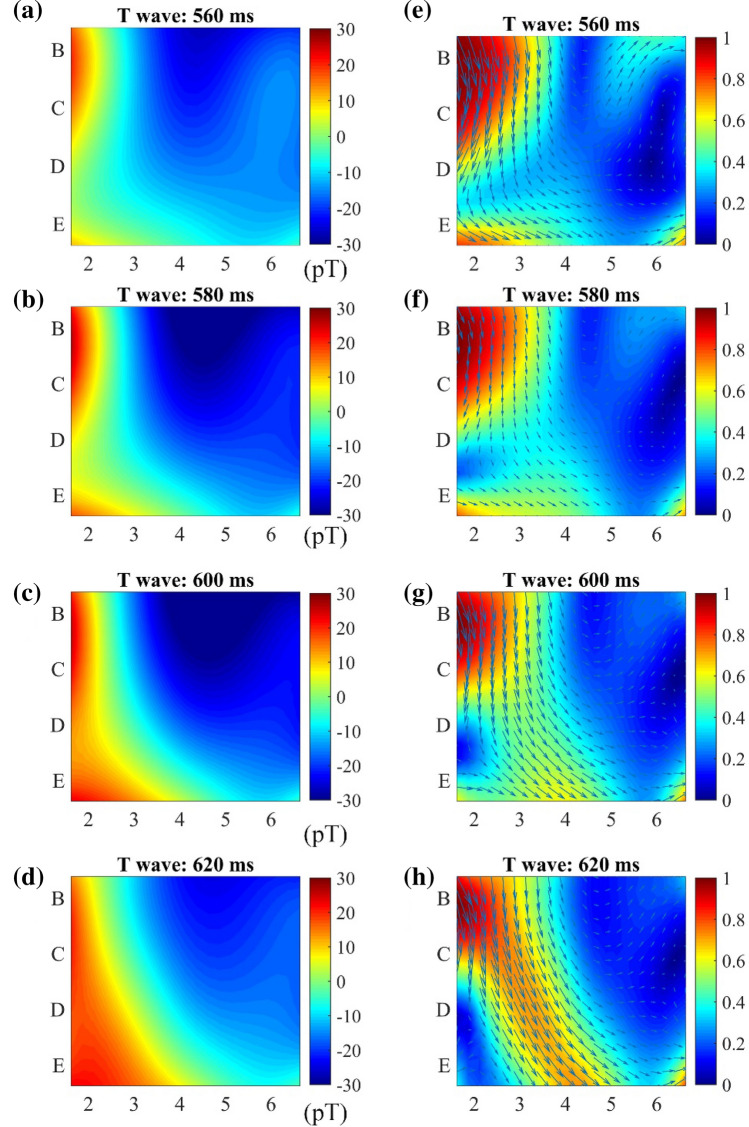
(**a**–**d**) MFMs and (**e**–**h**) corresponding PCD maps of a healthy subject during the T wave. The top to bottom panels represent the MFM and PCD maps constructed at different instants, 560 ms, 580 ms, 600 ms and 620 ms, of the cardiac cycle during the T-wave. (**e**–**h**) PCD map. The length and direction of the arrows indicate the magnitude and direction of the vector. In the MFM and PCD maps, both the Y-axis and X-axis scales represent sensor positions. The color of the MFM represents the magnetic field strength, and the color on the PCD maps represents the normalized current density.

Although the strongest signal occurs during the phase of the QRS complex, the T wave is of particular interest for cardiologists. Even with the cooccurring T wave in the ECG and MCG time series, the angular orientation of its corresponding current is only visible in the MCG 2D topography results, which has shown superior diagnostic value for the detection of coronary arterial diseases at rest^26^. To investigate the location of the cardiac current source during repolarization, MFMs and corresponding PCD maps during the T wave at different instants are shown in Fig. 9. It can be observed from a–d in Fig. 9 that the MFM always has a dipole structure at the T wave, and the dipole deflection angle is not large. The region in red represents the influx of the magnetic field, and the region in blue is the opposite. Meanwhile, the color depth indicates the strength of the magnetic field. In each PCD map, the direction and length of the arrow indicate the direction and magnitude of the underlying current flow. The magnitudes of the vectors are normalized using the min–max normalization method^27^. Thus, the color scales are graded between 0 (blue) and 1 (red), where blue represents the minimum current density and red represents the maximum current density. The region in deep red indicates the location of the maximum current flow source. The MFMs of healthy people have a very small deflection angle and appear as a dipole structure. For patients with heart disease, the deflection angle of MFMs during the T-wave period may vary greatly, and the direction of the current vector in the PCD maps is disordered.

## Discussion

In this study, a wearable multichannel human MCG system based on a SERF atomic magnetometer array is developed. The system consists of a magnetically shielded device, a wearable SERF magnetometer array, and a computer for data acquisition and processing. MCG signals on the body surface of a healthy subject are recorded. For cross validation, the ECG signal is recorded as a comparison. The results demonstrate the feasibility of the SERF atomic magnetometer for MCG measurement. To observe the temporal and spatial distribution of the human cardiac magnetic field, MFMs and PCD maps are obtained, by which the magnetic and current changes caused by cardiac activation can be visualized.

Our MCG system is wearable, which facilitates high-quality signal acquisition. Another advantage of the system is that simultaneous measurements will provide more spatial and temporal information. In addition, as the system operates at room temperature, the cost of this system is greatly reduced compared with traditional SQUID-based MCG systems.

However, the errors of position and orientation of magnetometers caused by the elasticity of the swimwear are not taken into account here, which will impact the multichannel MCG imaging results. A wireless motion tracking system produced by Polhemus Inc. is expected to provide both the position and orientation of the magnetometer.

## Methods

### Experimental setup

Single-channel MCG and ECG signals of the same position are first recorded to verify the measured MCG. Then, multichannel MCG experiments are carried out. Experiments were conducted to record MCG signals on the thoracic surface of a healthy male (age 23) who provided written informed consent (both to participate in the experiments and to release photographs). This project was reviewed and approved by the Ethics Committees of the Beijing Children’s Hospital, and all relevant ethical regulations on human experiments, including the Declaration of Helsinki, were followed. The risk of the subjects in this study was very small, and it was an observational study. There was no contact with the subjects, and no drugs were given to the subjects.

Before the experiments, 64 measurement points are uniformly marked on the swimwear, forming an 8 × 8 grid array, labelled A to G for rows and 1–8 for columns. The interval between adjacent points is 30 mm, covering an area of 210 mm × 210 mm over the chest, which is large enough to cover the four chambers of the human heart. Then, the receptacles are mounted across the holes on the marked points, as shown in Fig. 1b. The subject lays inside the magnetically shielded cylinder wearing swimwear with sockets, and then the magnetometers are plugged in the sockets perpendicular to the thoracic surface. The distance to the skin for each magnetometer is approximately 1 cm. In this study, only 20 SERF magnetometers are available. To obtain the cardiac magnetic field distribution on the whole thoracic surface of the subject simultaneously, 20-channel MCG signals at a 4 × 5 region were detected, covering the area of (B, C, D, E) × (2, 3, 4, 5, 6), which is close to the heart. The analog outputs of the 20 magnetometers are digitized at a sampling frequency of 1000 Hz.

### Signal component decomposition method

Independent component analysis (ICA)^28^ is one of the blind source separation techniques. This algorithm assumes that all the components are statistically independent of each other and that all of them are non-Gaussian distributed. According to the principle of statistical independence, the multichannel observation signals are decomposed into several independent components by the optimization algorithm. The observed signals X(t) are represented by a linear combination of the ICs S(t) as

1 $$X(t) = AS(t)$$

where $X(t) = [x_{1} (t),x_{2} (t), \cdots x_{m} (t)]^{T}$ is m observation vectors, $S(t) = [s_{1} (t),s_{2} (t), \cdots s_{n} (t)]^{T}$ is n source signal vectors $\left( {m \ge n} \right)$, and $A$ is the unknown mixing matrix. The observed signal is an instantaneous linear combination of unknown signal sources. The purpose of ICA is to estimate the independent sources and the mixed matrix as

2 $$\tilde{S}(t) = WX(t) = WAS(t)$$

where $W = A^{ - 1}$ is the unmixing matrix. The ICA method requires that the number of source components be less than or equal to the number of observed signals (or measured values). The mixed matrix A is the column full rank matrix. By ICA, several statistically independent components (IC) are extracted from the simultaneously measured multichannel signals, and the unknown mixing matrix is estimated. Multichannel signal noise elimination methods based on ICA have been widely used in recent years. After applying ICA, several noise-dominating ICs are eliminated, and the remaining signal-dominating ICs are further processed.

Empirical mode decomposition is an algorithm for analyzing time series signals, which decomposes them into several intrinsic mode function (IMF) amplitudes and frequency modulated zero-mean signals. Inspired by standard wavelet thresholding, a number of EMD-based denoising techniques have been developed^29^. In this study, the EMD interval thresholding (EMD-IT) method is used to eliminate the noise remaining in the signal-dominating ICs. The principle is that each IMF section is divided into several modal elements, and each modal element is taken as the processing object for thresholding. The absolute value of the extreme point is taken as the judgment standard. If the value is larger than the threshold, the unit is considered to be reserved as the main signal; otherwise, it is regarded as zero elimination of the noise unit. This method preserves complete modal elements, which can reduce the occurrence of discontinuities and enable IMF to have better continuity. After applying EMD-IT, the processed signal-dominating ICs are reprojected using the mixing matrix to obtain the denoised multichannel signals.

### MCG imaging

To visualize the distribution of the multichannel biomagnetic field, MFM has been widely used. In 1990, Schneider et al.^30^ developed an MCG imaging technique called cardiac magnetic field mapping by using a sensor array covering the whole chest. The visualization of biomagnetic measurement data by PCD maps using Hosaka–Cohen (HC) transformations became popular and was introduced by Cohen et al. in 1976^31^. The PCD map is considered a 2D presentation of a 3D current distribution and can provide an estimate of the underlying currents and their propagation^32^. The preprocessed data are interpolated using the cubic spline interpolation algorithm to generate smooth maps^33^. The current vector calculation formula of the PCD diagram is as follows:

3 $$\vec{I} = \frac{{\partial B_{Z} }}{\partial y}\vec{e}_{x} - \frac{{\partial B_{Z} }}{\partial x}\vec{e}_{y}$$

where $\vec{I}$ is the current vector in the measured plane, $B_{Z}$ represents the axial components of the measured cardiac magnetic field, and $\vec{e}_{x} ,\vec{e}_{y}$ are the unit direction vectors of $x$ and $y$ on the measurement plane, respectively. Its amplitude and phase angle are, respectively,

4 $$\left| I \right| = \sqrt {\left( {\frac{{\partial B_{Z} }}{\partial y}} \right)^{2} + \left( {\frac{{\partial B_{Z} }}{\partial x}} \right)^{2} }$$

5 $$\theta = \arctan \left( {{\raise0.7ex\hbox{${ - \frac{{\partial B_{Z} }}{\partial x}}$} \!\mathord{\left/ {\vphantom {{ - \frac{{\partial B_{Z} }}{\partial x}} {\frac{{\partial B_{Z} }}{\partial y}}}}\right.\kern-\nulldelimiterspace} \!\lower0.7ex\hbox{${\frac{{\partial B_{Z} }}{\partial y}}$}}} \right)$$

The PCD map is generated by PCD vectors calculated by the normal component of multichannel MCG signals and corresponding positions^31^. The amplitude and direction of vectors are reflected by the length and direction of arrows, forming a PCD map.
